# Awakening from Sleep and Hypoglycemia in Type 1 Diabetes Mellitus

**DOI:** 10.1371/journal.pmed.0040099

**Published:** 2007-02-27

**Authors:** Ilan Gabriely, Harry Shamoon

## Abstract

The authors discuss a new study showing that patients with type 1 DM are susceptible to nocturnal hypoglycemia and that there is a relationship between awakening and activation of the counterregulatory response to hypoglycemia.

## Impaired Counterregulation of Hypoglycemia in Type 1 Diabetes Mellitus

The Diabetes Control and Complications Trial in type 1 diabetes (T1DM) unequivocally showed the benefits of good glycemic control in preventing the complications of diabetes. Despite the treatment advances of the past decade, today iatrogenic hypoglycemia remains the major impediment to the appropriate control of blood glucose [1]. While imperfect insulin replacement places the patient at increased risk for frequent hypoglycemia, patients with T1DM also suffer from compromised counterregulatory responses to hypoglycemia [2]. The responses of all three main counterregulatory hormones normally responsible for rapid reversal of hypoglycemia are severely disrupted in T1DM. First, a decrease in plasma glucose cannot turn off endogenous insulin secretion (which is either insignificant or absent). Second, glucagon release during hypoglycemia is impaired soon after onset of diabetes. Third, epinephrine release during hypoglycemia becomes progressively defective in T1DM; its release is triggered only at lower plasma glucose levels, and the maximal concentration of epinephrine released is also significantly reduced [3]. This decrease in epinephrine response during hypoglycemia is accompanied by an attenuated autonomic neural response, which results in the clinical syndrome of impaired awareness of hypoglycemia (i.e., lack of the warning symptoms of prevailing hypoglycemia). Because of the disappearance of autonomic symptoms in such patients, mild hypoglycemia may occur without warning and may proceed unnoticed to more advanced and dangerous phases. Patients suffering from impaired awareness of hypoglycemia in addition to defective counterregulation may be at the greatest risk for developing severe hypoglycemia [4].

## Hypoglycemia-Associated Autonomic Failure

Hypoglycemia-associated autonomic failure (HAAF) describes a clinical syndrome apparently resulting from antecedent episodes of mild hypoglycemia that further degrade the counterregulatory response. As shown experimentally in people without diabetes, recurrent and/or recent episodes of hypoglycemia are associated with reduced sympathoadrenal (epinephrine and norepinephrine), symptomatic, and cognitive responses to subsequent hypoglycemia, impairing all of the defense mechanisms required for prevention and reversal of hypoglycemia [5]. Since patients with T1DM have an already reduced counterregulatory response (as mentioned above), HAAF represents a vicious cycle of hypoglycemia begetting further—and more severe—hypoglycemia. The critical role played by even mild episodes of hypoglycemia is exemplified by studies that show that avoidance of hypoglycemia can improve the epinephrine response and reverse impaired awareness of hypoglycemia [6]. Furthermore, recent studies have shown improvement in the counterregulatory response to hypoglycemia in T1DM using pharmacological interventions, suggesting that hypoglycemia sensing plays a role in HAAF [7–9].

## Nocturnal Hypoglycemia

A particularly important condition, observed mainly in T1DM, is nocturnal hypoglycemia, which may be asymptomatic and often is neither suspected nor recognized. Plasma glucose is rarely measured during sleep, and nocturnal hypoglycemia may therefore not be confirmed. Factors that contribute to the development of nocturnal hypoglycemia include preceding physical activity; imbalance between the insulin regimen and the amount, content, and timing of meals; and alcohol consumption. In addition, there may be enhanced sensitivity to insulin, and sleep per se is associated with a decrease in the autonomic response to hypoglycemia [10]. Although most episodes of nocturnal hypoglycemia are asymptomatic, some patients complain of sleep disturbances (vivid dreams or nightmares), morning headache (a “hung-over” sensation), chronic fatigue, or mood changes (mainly depression). As noted above, recurrent episodes of hypoglycemia cause further deterioration of the counterregulatory response to subsequent hypoglycemia. Thus, nocturnal hypoglycemia may be an important factor in precipitating daily hypoglycemia episodes and exacerbating HAAF [11].

## Awakening from Sleep with Hypoglycemia

Although the clinical problem of nocturnal hypoglycemia in T1DM is well recognized, less is known about the mechanisms responsible for awakening during episodes of nocturnal hypoglycemia, and their perturbation in T1DM. Understanding these mechanisms is particularly important given data on the “dead in bed” syndrome, hypothesized to be due to a combination of undetected autonomic dysfunction and nocturnal hypoglycemia that may predispose T1DM patients to fatal ventricular dysrhythmias [12]. Moreover, probably owing to reduced sympathoadrenal responses, patients with T1DM are less likely to be awakened by hypoglycemia [13].

Banarer et al. [14] have shown that patients with T1DM are substantially less likely to be awakened by hypoglycemia, and that their autonomic responses to hypoglycemia are reduced during sleep. In their studies, however, hypoglycemia was induced before the patients were asleep.

In order to better define the relationship between nocturnal hypoglycemia and awakening from sleep, Bernd Schultes and colleagues studied 16 patients with T1DM and 16 healthy controls in two experimental conditions: (1) insulin-induced hypoglycemia (40 mg/dl) during sleep together with polysomnographic measurements and serial determination of the hormonal counterregulatory response, and (2) the same experimental protocol, but with maintenance of euglycemia. The researchers now report their findings in *PLoS Medicine* [15].

The goal of the study was to determine the occurrence of awakening during the period of hypoglycemia and its correlation to the hormonal counterregulatory response. There were three important findings. First, only one of the 16 patients with T1DM had spontaneous awakening during hypoglycemia, whereas ten of the 16 healthy controls were awakened by hypoglycemia. Second, the hormonal counterregulatory response to hypoglycemia was (as expected) diminished in the patients with T1DM. Finally, in all cases of awakening during hypoglycemia, there was an associated increase in plasma epinephrine and cortisol, suggesting a relationship between the counterregulatory response (i.e., recovery of low blood glucose levels) and awakening from sleep.

## Clinical Implications of Nocturnal Hypoglycemia in T1DM

Prevention of nocturnal hypoglycemia involves several strategies, including “fine tuning” of the insulin regimen, the use of “long-acting” bedtime snacks, routine monitoring of blood glucose at bedtime and before breakfast, and occasional monitoring of glucose at the time of maximum risk during sleep [16]. The development of continuous noninvasive glucose-monitoring devices is a promising strategy. Not only can such devices produce a continuous profile of blood glucose levels in order to better adjust treatment, but they also include audible alarms that can awaken the patient before the onset of dangerous hypoglycemia.

Schultes and colleagues' study [15] strongly supports and further advances the current notion of T1DM susceptibility to nocturnal hypoglycemia and shows a clear relationship between awakening and activation of the counterregulatory response to hypoglycemia, although a cause–effect could not be established with this study design. Also, because of the rapid onset of hypoglycemia, glycemic thresholds for awakening could not be determined with precision, and no firm associations with specific counterregulatory hormones could be made.

Additional studies are needed to examine whether the abnormal autonomic response in T1DM is further impaired during sleep, and whether a direct correlation can be established between the degree of counterregulation impairment (or HAAF) and nocturnal awakening during hypoglycemia; both factors could play important roles in the susceptibility to nocturnal hypoglycemia in T1DM.

## Abbreviations

HAAF: hypoglycemia-associated autonomic failure
T1DM: type 1 diabetes mellitus
